# Identification of TMEM41B TM6 as a critical domain for coronavirus replication *in vitro* and *in vivo*

**DOI:** 10.1128/jvi.00493-26

**Published:** 2026-07-24

**Authors:** Puxian Fang, Wenwen Xiao, Xunlei Wang, Yuanxiang Xiong, Tong Ding, Ting Cheng, Chaoqun Chen, Shaobo Xiao, Liurong Fang

**Affiliations:** 1 National Key Laboratory of Agricultural Microbiology, College of Veterinary Medicine, Huazhong Agricultural University 47895, Wuhan, China; 2 College of Veterinary Medicine, Shandong Agricultural University, Taian, China; 3 Shandong Provincial Key Laboratory of Zoonoses, Shandong Agricultural University 34734, Taian, China; 4 Key Laboratory of Preventive Veterinary Medicine in Hubei Province, The Cooperative Innovation Center for Sustainable Pig Production, Wuhan, China; University of Michigan Medical School, Ann Arbor, Michigan, USA

**Keywords:** coronaviruses, porcine deltacoronavirus (PDCoV), TMEM41B, sixth transmembrane domain (TM6), antiviral gene-edited animals

## Abstract

Transmembrane protein 41B (TMEM41B) serves as a critical host factor for various coronavirus infections, providing a potential target for developing antiviral gene-edited animals. However, TMEM41B is a gene essential for mammalian embryonic development, with complete knockout resulting in embryonic lethality. Consequently, it is of paramount importance to identify its critical functional domains that are amenable to deletion. In this study, we demonstrated that TMEM41B is involved in the replication stage of porcine deltacoronavirus (PDCoV), an emerging zoonotic coronavirus that infects various animals and even humans, but not in the attachment, internalization, or release steps. Through the construction of truncated mutants and cell lines stably expressing or precisely lacking the functional domains of TMEM41B, we confirm that, in addition to the VTT domain, the sixth transmembrane (TM6) domain of TMEM41B is also required for coronavirus infection. Using CRISPR/Cas9 technology, we generated a C57BL/6J mouse model with a deletion of the TMEM41B TM6 domain. Animal experiments demonstrated that the deletion of the TM6 domain significantly inhibited viral infection *in vivo* and mitigated liver tissue damage caused by mouse hepatitis virus (MHV), thereby increasing the survival rate of mice after MHV infection. Taken together, this study is the first to report a requirement of TMEM41B TM6 domain for coronavirus infection, deepening our understanding of TMEM41B function and providing novel insights for the development of antiviral gene-edited animals.

**IMPORTANCE:**

TMEM41B is a proviral host factor for pan-coronaviruses, making it an ideal target for developing antiviral gene-edited animals. However, the essential role of TMEM41B in mammalian embryonic development poses a major obstacle to this goal. Identifying its critical functional domains or key amino acids that are amenable to deletion is pivotal for evaluating TMEM41B as a viable target for developing antiviral gene-edited animals. Our present study demonstrates that the sixth transmembrane (TM6) domain of TMEM41B is critical for the infection of PDCoV and other coronaviruses, without obviously compromising the function of its conserved VTT domain. Furthermore, a mouse infection model provides strong evidence that deletion of TM6 suppresses viral infection and attenuates virus-induced liver damage. Collectively, we identify TM6 as a crucial domain for coronavirus infection both *in vitro* and *in vivo*, providing new insights into the structure-function relationship of TMEM41B and proposing a novel strategy for antiviral animal development.

## INTRODUCTION

Coronaviruses (CoVs) are enveloped positive-strand RNA viruses that belong to the subfamily *Coronavirinae* within the family *Coronaviridae* of the order *Nidovirales*. They are classified into four genera: *Alphacoronavirus* (α-CoV), *Betacoronavirus* (β-CoV), *Gammacoronavirus* (γ-CoV), and *Deltacoronavirus* (δ-CoV) (1, 2). CoV infections usually cause various diseases, from mild respiratory symptoms, gastroenteritis, and hepatitis to severe outcomes, including death in both animals and humans (3). The α-CoVs and β-CoVs exclusively infect mammals, such as α-CoVs, which include the porcine enteric diarrhea virus (PEDV), porcine transmissible gastroenteritis virus (TGEV), and human coronaviruses 229E (HCoV-229E) and OC43 (HCoV-OC43), as well as β-CoVs, which include mouse hepatitis virus (MHV), severe acute respiratory syndrome coronavirus (SARS-CoV), Middle East respiratory syndrome coronavirus (MERS-CoV), and SARS-CoV-2 (4, 5). The most well-studied γ-CoV is the infectious bronchitis virus, which primarily infects birds (6). δ-CoVs can infect both mammals and birds, including bulbul coronavirus and porcine deltacoronavirus (PDCoV) (1, 7–9). Since PDCoV was first reported to cause outbreaks in pig farms in Ohio, USA, in early 2014, it has rapidly spread to many countries and regions (8, 10–13). Notably, more than 20 types of cells are susceptible to PDCoV *in vitro*, and PDCoV has been experimentally confirmed to have broad host tropism, including calves (14), turkeys (15), chickens (16), mice (17, 18), ducks, geese (19), and ferrets (20). Importantly, three cases of natural infection in Haitian children were reported (21). Therefore, PDCoV has been proposed as the eighth coronavirus with the ability of cross-species transmission (22), posing a significant threat to both human and animal health.

The establishment of effective viral infection is highly dependent on host factors. However, the key host factors required for the infection of coronaviruses remain underexplored. CRISPR-based screening serves as a powerful tool for systematically investigating host factors involved in viral infection and has been extensively applied to SARS-CoV-2 (23–25), Japanese encephalitis virus (26), influenza A virus (27), PEDV, and PDCoV (28). Among the identified host factors, transmembrane protein 41B (TMEM41B) has emerged as a significant contributor, playing a crucial role in promoting infection by coronaviruses and three additional virus families (23, 24, 29, 30), suggesting that TMEM41B is a potential target for therapeutic intervention. To date, the underlying mechanisms and the key functional domains of TMEM41B that facilitate viral replication remain poorly understood and warrant further investigation. Previous studies have shown that TMEM41B is an integral membrane protein of the endoplasmic reticulum (ER) and regulates the formation of autophagosomes, lipid droplets (LDs), and lipoproteins (31–34), making it an essential gene for mammalian embryonic development (35). TMEM41B contains a critical VTT domain, and a recent study has demonstrated that the TMEM41B VTT domain is necessary for supporting flavivirus infection (30). However, whether the function of the VTT domain of TMEM41B is universal across other virus families and whether other domains of TMEM41B are required for its proviral function remain largely unclear.

In this study, we demonstrated that TMEM41B is involved in the replication step of PDCoV infection. Our results also indicate that, similar to the VTT domain, the sixth transmembrane region (TM6) of TMEM41B is equally vital for the infection of various coronaviruses. Furthermore, a mouse infection model confirmed that the deletion of TM6 significantly mitigated the liver tissue damage and reduced viral titers associated with MHV infection *in vivo*. These results highlight the significance of TMEM41B TM6 in coronavirus infection and provide new insights for the development of anticoronavirus gene-edited animals.

## RESULTS

### TMEM41B knockout inhibits coronavirus infection

**Fig 1:**
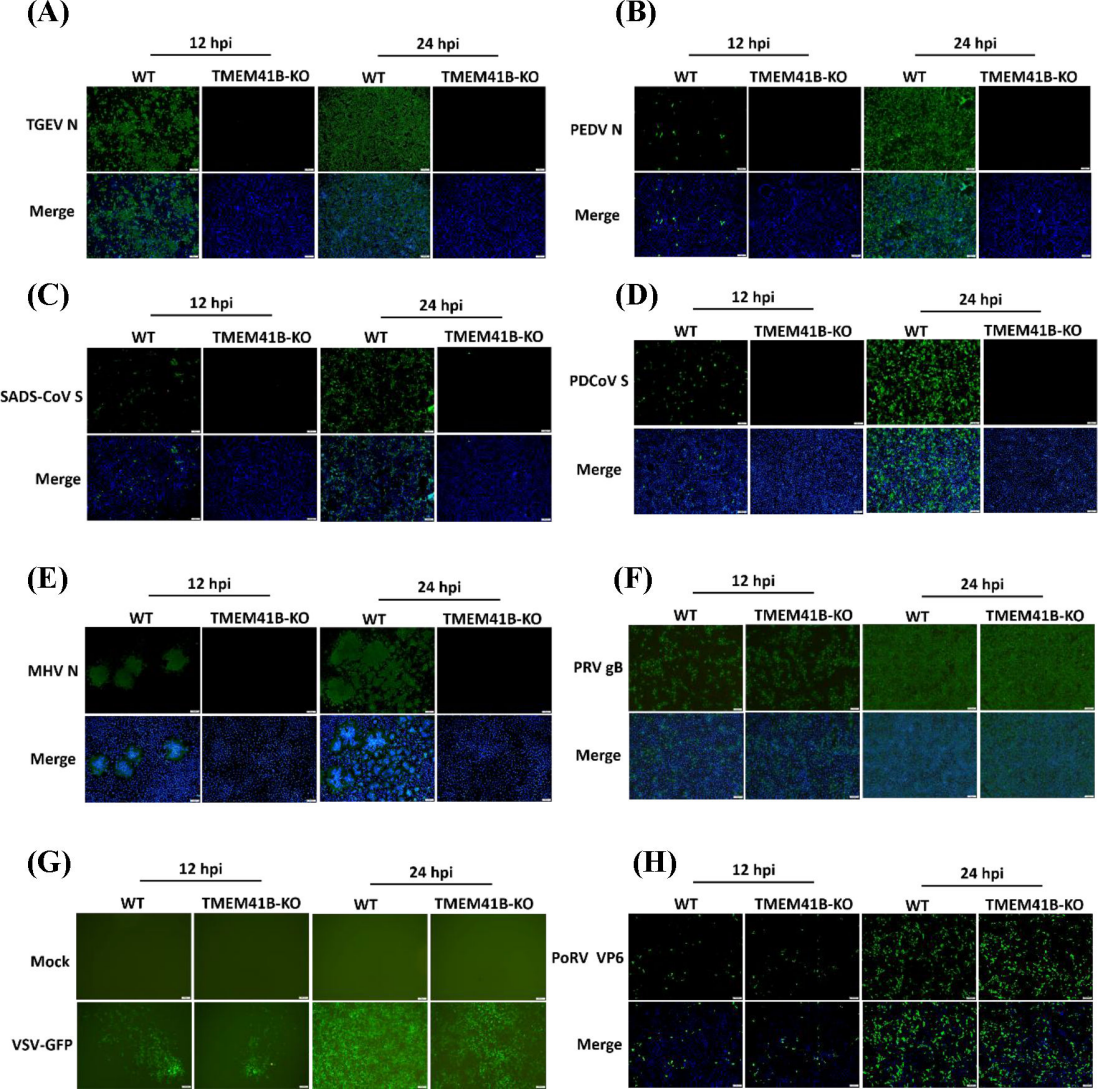
TMEM41B knockout inhibits coronavirus infection. (**A–D**) WT and TMEM41B-KO LLC-PK1 cells were infected with TGEV, PDCoV (multiplicity of infection [MOI] of 0.1), PEDV, and SADS-CoV (MOI = 1) for 24 h, followed by IFA for detecting viral infection. (**E**) WT and TMEM41B-KO L929 cells were infected with MHV-A59 (MOI = 0.01). After 24 hpi, cells were harvested and subjected to IFA. (**F–H**) WT and TMEM41B-KO LLC-PK1 cells were infected with PRV, VSV-GFP (MOI = 1), and PoRV (MOI = 10) for 24 h, followed by IFA. Scale bar, 100 μm.

Several studies have shown that TMEM41B functions as a proviral host factor for a variety of coronaviruses, such as SARS-CoV-2, MERS-CoV, HCoV-229E, and HCoV-OC43 (24, 29, 36). Here, we generated a TMEM41B-knockout (TMEM41B-KO) LLC-PK1 cell line and investigated whether TMEM41B knockout affects the infection of several other swine enteropathogenic coronaviruses, including TGEV, PEDV, swine acute diarrhea syndrome coronavirus (SADS-CoV), and PDCoV. The wild-type (WT) and TMEM41B-KO LLC-PK1 cells were infected with TGEV, PEDV, SADS-CoV, and PDCoV, respectively, followed by indirect immunofluorescence assay (IFA) at 12 and 24 h post-infection (hpi). The results showed that specific green fluorescence was observed in WT LLC-PK1 cells infected with TGEV, PEDV, SADS-CoV, or PDCoV. In contrast, there was no green fluorescence in TMEM41B-KO LLC-PK1 cells following infection with the corresponding viruses (Fig. 1A through D). Similar results were also observed in the TMEM41B-KO L929 cells infected with MHV (Fig. 1E). Additionally, we assessed the infection of pseudorabies virus (PRV, a double-strand DNA virus), vesicular stomatitis virus (VSV, a negative-strand RNA virus), and porcine rotavirus (PoRV, a double-stranded RNA virus) in both WT and TMEM41B-KO LLC-PK1 cells. The results showed that green fluorescence exhibited no obvious differences between WT and TMEM41B-KO LLC-PK1 cells during infections with PRV, VSV-GFP, and PoRV (Fig. 1F through H). Collectively, these results further substantiate that the knockout of TMEM41B impedes coronavirus infection.

### TMEM41B functions at a post-entry step of the PDCoV replication cycle

**Fig 2:**
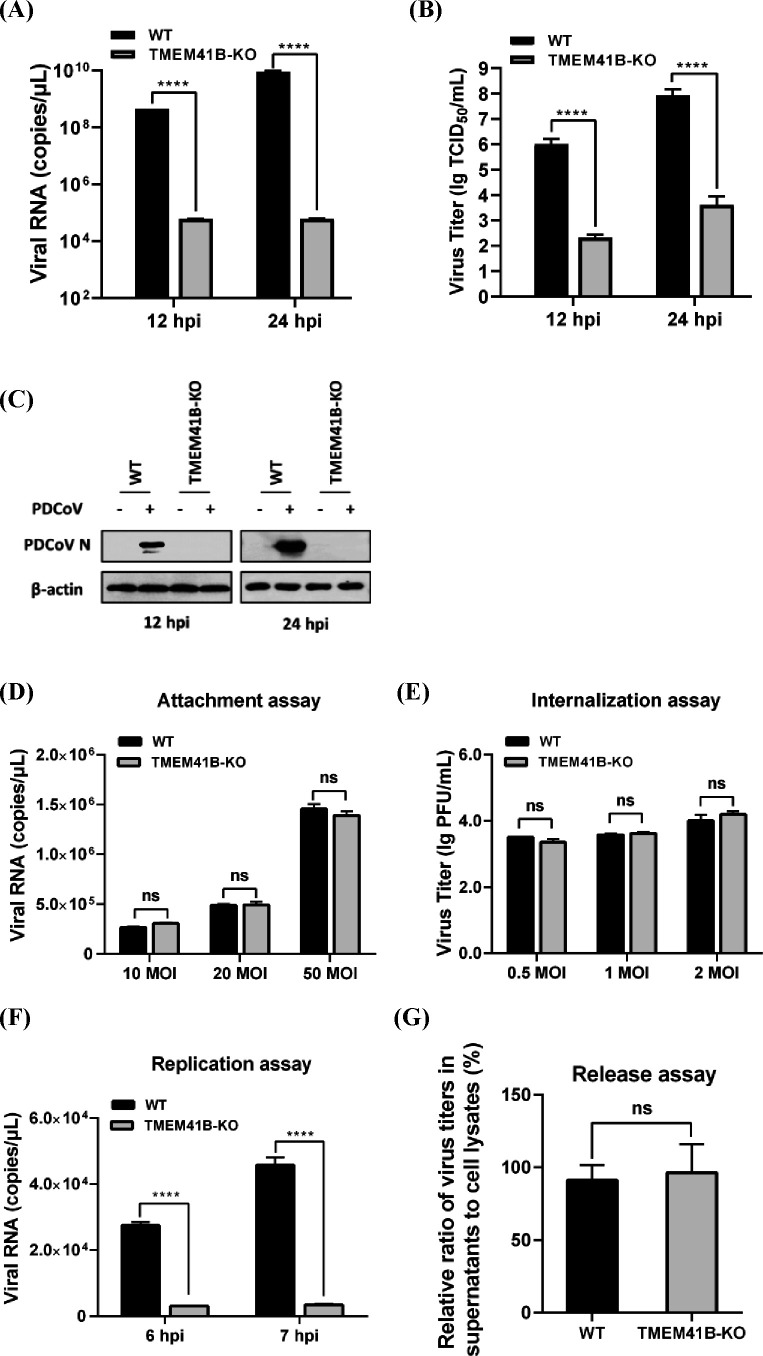
TMEM41B functions at a post-entry step of the PDCoV replication cycle. (**A–C**) PDCoV infection was analyzed in WT and TMEM41B-KO LLC-PK1 cells via RT-qPCR (**A**), TCID_50_ (**B**), and Western blot assays (**C**). WT and TMEM41B-KO LLC-PK1 cells were infected with PDCoV (MOI = 0.1). After 12 and 24 hpi, cells were collected and subjected to the detection of viral infection. Scale bar, 100 μm. (**D**) WT and TMEM41B-KO LLC-PK1 cells were infected with increasing amounts of PDCoV (MOI = 10, 20, 50) at 4°C for 1 h. Cells were washed with citrate buffer (pH = 3) and collected for RT-qPCR. (**E**) WT and TMEM41B-KO LLC-PK1 cells were infected with increasing amounts of PDCoV (MOI = 0.5, 1, 2) at 4°C for 1 h. After three washes with citrate buffer, cells were cultured at 37°C for 1 h, followed by cellular sample collection and plaque assays in WT LLC-PK1 cells. (**F**) WT and TMEM41B-KO LLC-PK1 cells were infected with PDCoV (MOI = 0.5) at 4°C for 1 h. After three washes with citrate buffer, cells were cultured at 37°C. After 6 and 7 hpi, cells were collected for RT-qPCR. (**G**) WT and TMEM41B-KO LLC-PK1 cells were infected with PDCoV for 12 h, and cellular supernatants were removed, followed by the addition of fresh MEM with 7.5 μg/mL trypsin. After 1 hpi, cellular supernatants and cells were collected for TCID_50_ assay, respectively. The results present the relative ratio of virus titers in supernatants and cell lysates. ****P < 0.0001.

We used PDCoV as a representative to further investigate the detailed role of TMEM41B in coronavirus infection. WT and TMEM41B-KO LLC-PK1 cells were infected with PDCoV at different time points, followed by the detection of viral infection through reverse transcription quantitative real-time PCR (RT-qPCR), TCID_50_ and Western blot assays. The results from the RT-qPCR and TCID_50_ assays showed that TMEM41B knockout significantly reduced viral RNA levels and titers (Fig. 2A and B). Consistent with the above results, the Western blot assay showed a markedly reduced expression of PDCoV N protein in TMEM41B-KO LLC-PK1 cells compared to WT LLC-PK1 cells (Fig. 2C). These results suggest that TMEM41B knockout suppresses PDCoV infection. To further identify which stages of the PDCoV replication cycle are affected by TMEM41B knockout, we performed viral attachment, internalization, replication, and release assays in WT and TMEM41B-KO LLC-PK1 cells. The results revealed that TMEM41B knockout significantly inhibited the viral replication step while not affecting the attachment, internalization, and release steps (Fig. 2D through G). Taken together, these results indicate that TMEM41B knockout inhibits PDCoV infection at a stage subsequent to viral entry into the cells.

### TMEM41B-mediated PDCoV infection depends on its VTT and TM6 domains

**Fig 3:**
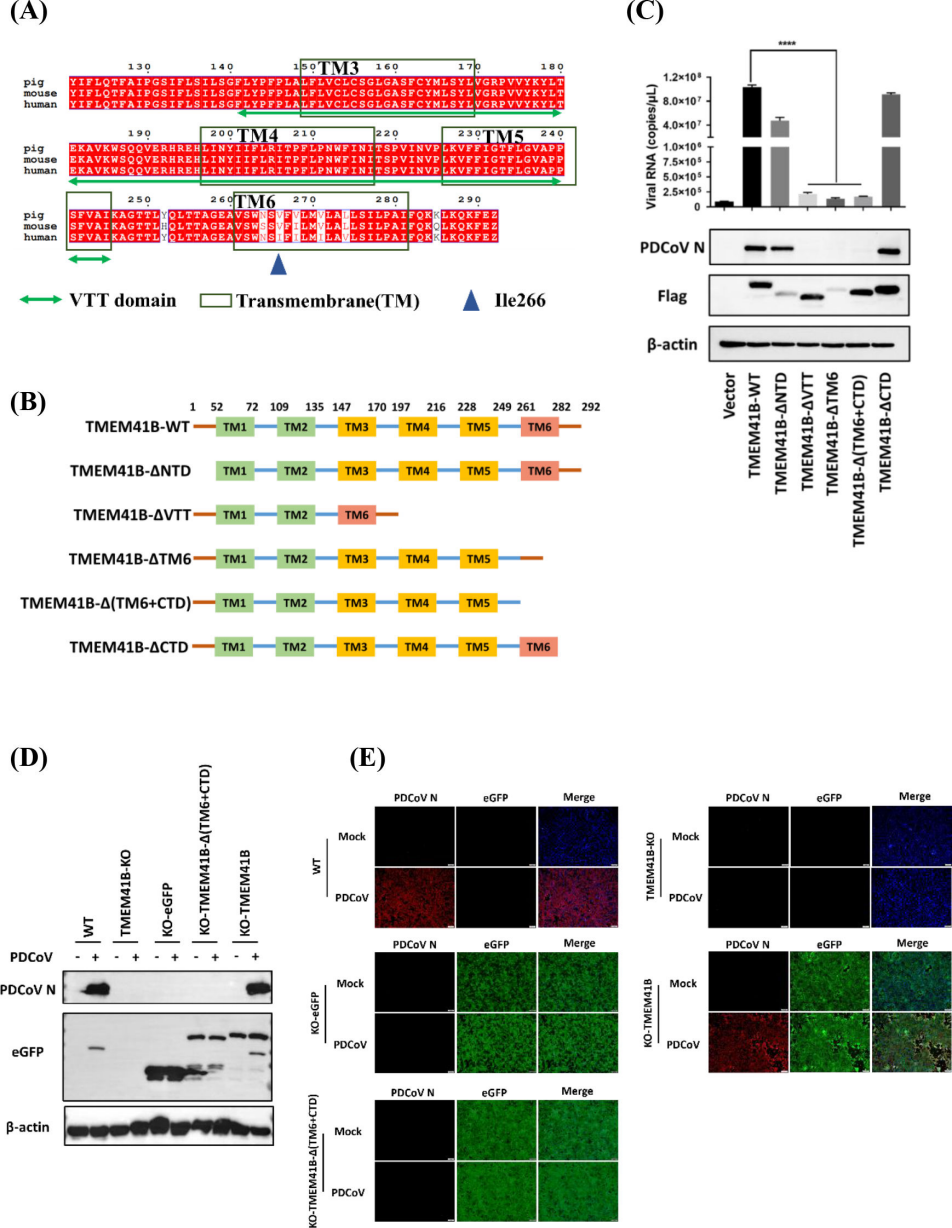
TMEM41B-mediated PDCoV infection depends on its VTT and TM6 domains. (**A**) Amino acid alignment analyses for mouse, pig, and human TMEM41B. (**B**) Schematic diagram of construction of truncated TMEM41B mutants. (**C**) TMEM41B-KO LLC-PK1 cells were transfected with TMEM41B-WT, TMEM41B mutant expression plasmids encoding TMEM41B-ΔNTD, -ΔVTT, -ΔTM6, -Δ(TM6+CTD), -ΔCTD, or the empty vector for 24 h, and then infected with PDCoV (MOI = 0.1). After 24 hpi, cells were collected for Western blot. (**D and E**) Three LLC-PK1 cells stably expressing eGFP, TMEM41B-Δ(TM6+CTD), or TMEM41B were infected with PDCoV for 12 h, followed by Western blot (**D**) and IFA (**E**). WT and TMEM41B-KO LLC-PK1 cells were used as positive and negative controls, respectively. Scale bar, 100 μm. ****P < 0.0001.

TMEM41B is a multi-transmembrane protein localized in the ER, characterized by a core that contains a VTT domain, comprising transmembrane segments TM3, TM4, and TM5 (33). A previous study has demonstrated that mutation in TMEM41B Ile266 attenuates flavivirus infection in cell culture, potentially explaining the variability in virus infection among different human populations (30). To further investigate the amino acid homology of TMEM41B across various species, we conducted an alignment analysis of TMEM41B from porcine, mouse, and human sources. The results revealed a high degree of amino acid identity among porcine, mouse, and human TMEM41B, particularly within the VTT domain (Fig. 3A). However, we found that the Ile266 residue in human TMEM41B is situated outside the VTT domain, specifically within TM6 (Fig. 3A). To further determine which domains of TMEM41B affect viral infection, we constructed a series of eukaryotic expression plasmids encoding the full-length TMEM41B (designated TMEM41B-WT) and truncated mutants lacking individual domains: NTD, VTT, TM6, TM6+CTD, or CTD, referred to as TMEM41B-ΔNTD, -ΔVTT, -ΔTM6, -Δ(TM6+CTD), and -ΔCTD, respectively (Fig. 3B). TMEM41B-WT and its mutants were transfected into TMEM41B-KO LLC-PK1 cells, followed by PDCoV infection and subsequent detection of viral replication. As shown in Fig. 3C, TMEM41B-WT, as well as the truncated mutants TMEM41B-ΔNTD and TMEM41B-ΔCTD, could obviously restore PDCoV infection compared to the control group. Conversely, the mutants TMEM41B-ΔVTT, -ΔTM6, and -Δ(TM6+CTD) did not efficiently support PDCoV infection. Given that the expression level of TMEM41B-ΔTM6 is lower than that of TMEM41B-WT, it remains a possibility that its inability to support PDCoV infection results from reduced protein expression. However, TMEM41B-Δ(TM6+CTD) and -ΔCTD mutants exhibited comparable protein expression levels to TMEM41B-WT. Notably, TMEM41B-ΔCTD effectively supported PDCoV infection, while TMEM41B-Δ(TM6+CTD) did not, underscoring the critical role of TM6 in PDCoV infection.

**Fig 4:**
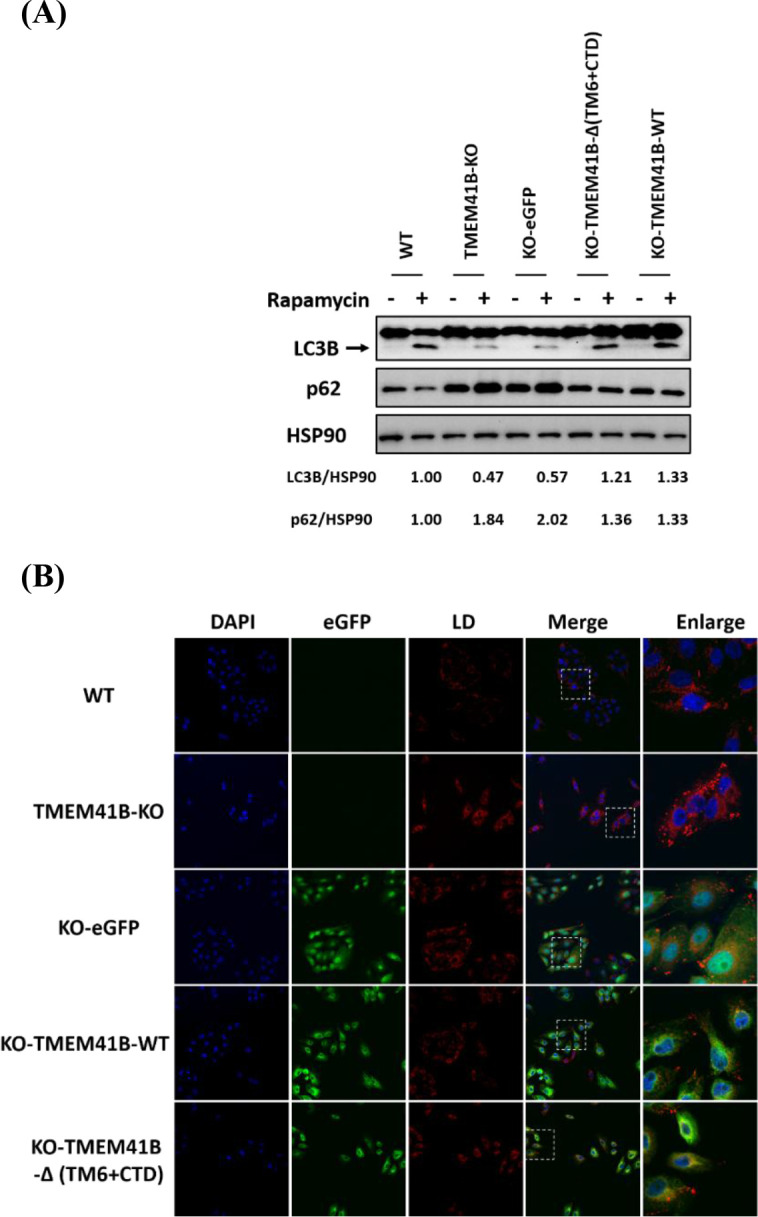
TMEM41B-Δ(TM6+CTD) retains the ability to maintain the autophagy process and LD homeostasis *in vitro*. (**A**) WT, TMEM41B-KO LLC-PK1, and three LLC-PK1 cells stably expressing eGFP, TMEM41B-WT, or TMEM41B-Δ(TM6+CTD) were treated with rapamycin or left untreated, followed by detecting autophagic markers LC3B and p62 via Western blot. The numbers below the images represent the relative level of LC3B/p62 to HSP90 via ImageJ software analysis. (**B**) HCS LipidTOX Deep Red neutral lipid stain was used to detect the lipid distribution in three LLC-PK1 cells individually expressing eGFP, TMEM41B-WT, and TMEM41B-Δ(TM6+CTD). WT and TMEM41B-KO LLC-PK1 cells were used as controls.

To further confirm this conclusion and ensure consistency with the results obtained from *in vitro* overexpression, we constructed three TMEM41B-KO LLC-PK1 cell lines stably expressing eGFP (as a control), TMEM41B-WT, and TMEM41B-Δ(TM6+CTD), designated as KO-eGFP, KO-TMEM41B-WT, and KO-TMEM41B-Δ(TM6+CTD), respectively (Fig. S1A and B). Subsequently, PDCoV infections were analyzed in these cell lines, as well as in WT or TMEM41B-KO LLC-PK1 cells (as additional controls). As expected, Western blot and IFA results showed that PDCoV established efficient infection only in WT LLC-PK1 cells and TMEM41B-KO LLC-PK1 cells stably expressing TMEM41B-WT (Fig. 3D and E). Additionally, we detected the autophagy pathway and LD homeostasis in these cell lines and found that the TMEM41B-KO and KO-eGFP cell lines exhibited accumulations of p62 and intracellular LDs, indicating defects in both the autophagic degradation process and LD metabolism (Fig. 4A and B). Conversely, both KO-TMEM41B-Δ(TM6+CTD) and KO-TMEM41B-WT cells, similar to WT LLC-PK1 cells, did not show abnormal accumulations of p62 and LDs (Fig. 4A and B), demonstrating that the TM6 domain deletion does not significantly impact autophagy regulation and LD homeostasis *in vitro*. Taken together, these results indicate that, in addition to the VTT domain, the TM6 domain is also required for TMEM41B to promote PDCoV replication.

### Deletion of TMEM41B TM6 fails to support coronavirus infection

**Fig 5:**
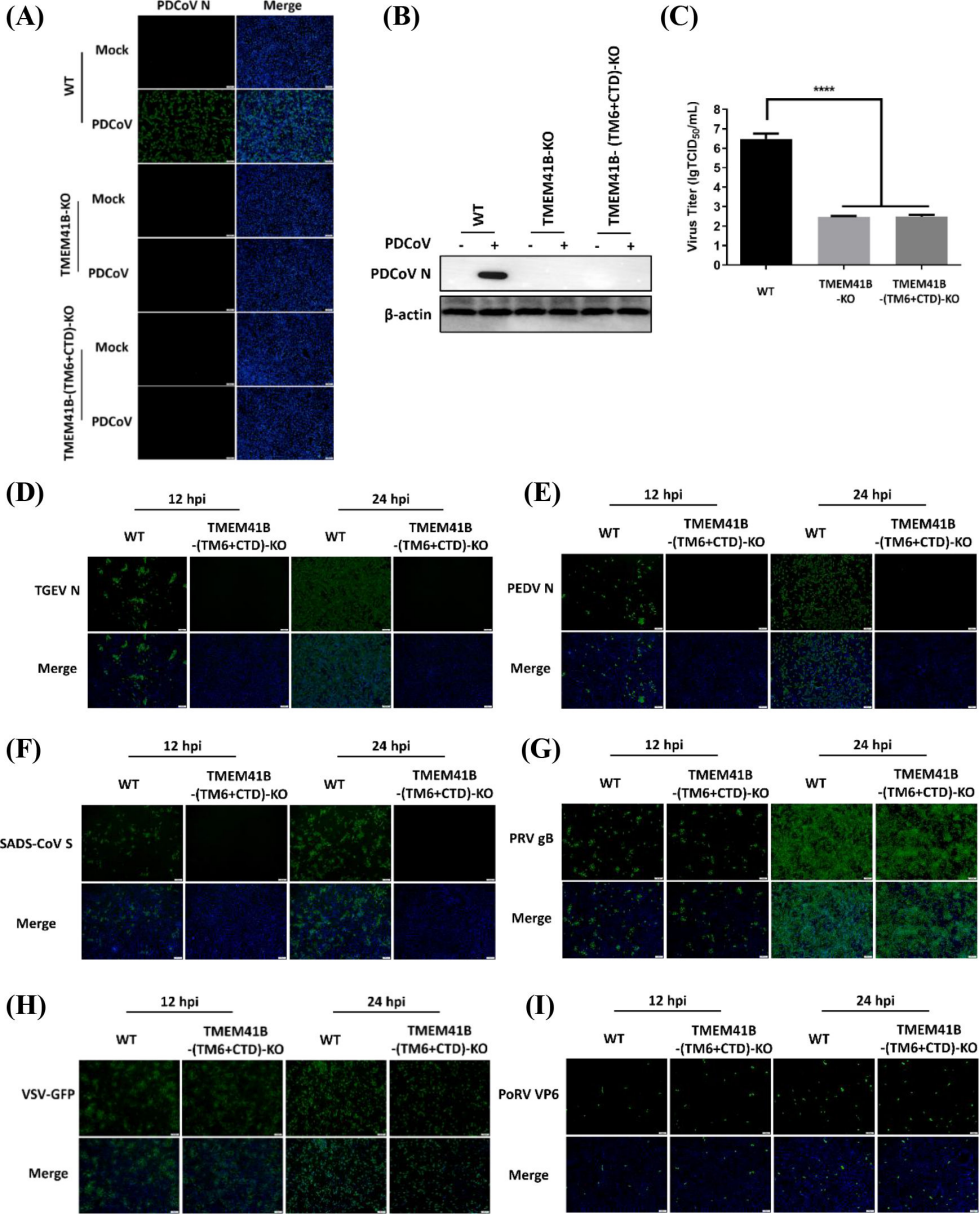
Deletion of TMEM41B TM6 domain fails to support coronavirus infection. (**A–C**) TMEM41B-(TM6+CTD)-KO LLC-PK1 cells were infected with PDCoV (MOI = 0.1) for 12 h, followed by IFA (**A**), Western blot (**B**), and TCID_50_ (**C**) assays. WT and TMEM41B-KO LLC-PK1 cells were used as positive and negative controls, respectively. (**D–I**) WT and TMEM41B-(TM6+CTD)-KO LLC-PK1 cells were infected with different viruses, including TGEV, PEDV, SADS-CoV, PRV, VSV-GFP, and PoRV. After 12 and 24 hpi, cells were collected for IFA. Scale bar, 100 μm. ****P < 0.0001.

To further confirm the role of the TM6 domain in coronavirus infection, we constructed two cell lines, LLC-PK1 (Fig. S2A and C) and L929 (Fig. S2B and C) with precise deletions of TMEM41B TM6+CTD, designated TMEM41B-(TM6+CTD)-KO. The infection of PDCoV was examined in TMEM41B-(TM6+CTD)-KO LLC-PK1 cells using IFA, Western blot, and TCID_50_ assays. As shown in Fig. 5A and B, specific green fluorescence and PDCoV N protein were detected in WT LLC-PK1 cells but not in TMEM41B-KO and TMEM41B (TM6+CTD)-KO LLC-PK1 cells. Consistent with the results from IFA and Western blot, the TCID_50_ assay showed that viral titers in TMEM41B-(TM6+CTD)-KO and TMEM41B-KO LLC-PK1 cells were significantly lower than those in WT LLC-PK1 cells (Fig. 5C). In addition, TMEM41B and TMEM41B TM6+CTD knockout had no obvious negative effects on baseline cell proliferation (Fig. S3).

**Fig 6:**
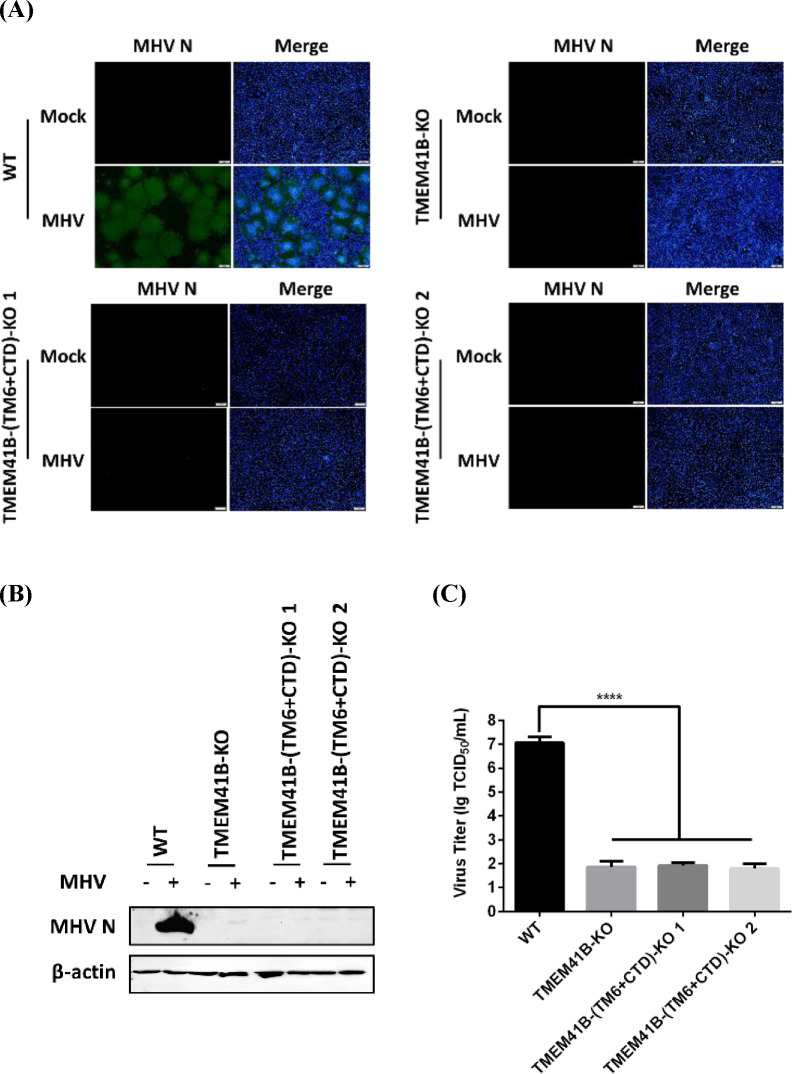
TMEM41B TM6 domain deletion inhibits MHV infection. (**A–C**) WT, TMEM41B-KO, and TMEM41B-(TM6+CTD)-KO L929 cells were infected with MHV-A59 for 12 h, respectively. Cells were harvested and subjected to IFA (**A**), Western blot (**B**), and TCID_50_ (**C**) assays. WT and TMEM41B-KO L929 cells were used as positive and negative controls, respectively. Scale bar, 100 μm. ****P < 0.0001.

We further detected the infection of other swine enteric coronaviruses (TGEV, PEDV, and SADS-CoV), as well as PRV, VSV-GFP, and PoRV in WT and TMEM41B-(TM6+CTD)-KO LLC-PK1 cells at 12 and 24 hpi. The IFA results showed that TGEV, PEDV, and SADS-CoV could not establish efficient infection in TMEM41B-(TM6+CTD)-KO LLC-PK1 cells, as demonstrated by the absence of specific green fluorescence (Fig. 5D through F). In contrast, the infections of PRV, VSV-GFP, and PoRV showed no obvious differences between WT and TMEM41B-(TM6+CTD)-KO LLC-PK1 cells (Fig. 5G through I). Additionally, MHV infection in TMEM41B-(TM6+CTD)-KO and TMEM41B-KO L929 cells was significantly lower than that in WT L929 cells, as determined by IFA, Western blot, and TCID_50_ assays (Fig. 6A through C). These results indicate that the TMEM41B TM6 domain is required for coronavirus infection *in vitro* and may represent a potential target for genetic editing.

### TMEM41B TM6 domain is required for coronavirus infection *in vivo*

**Fig 7:**
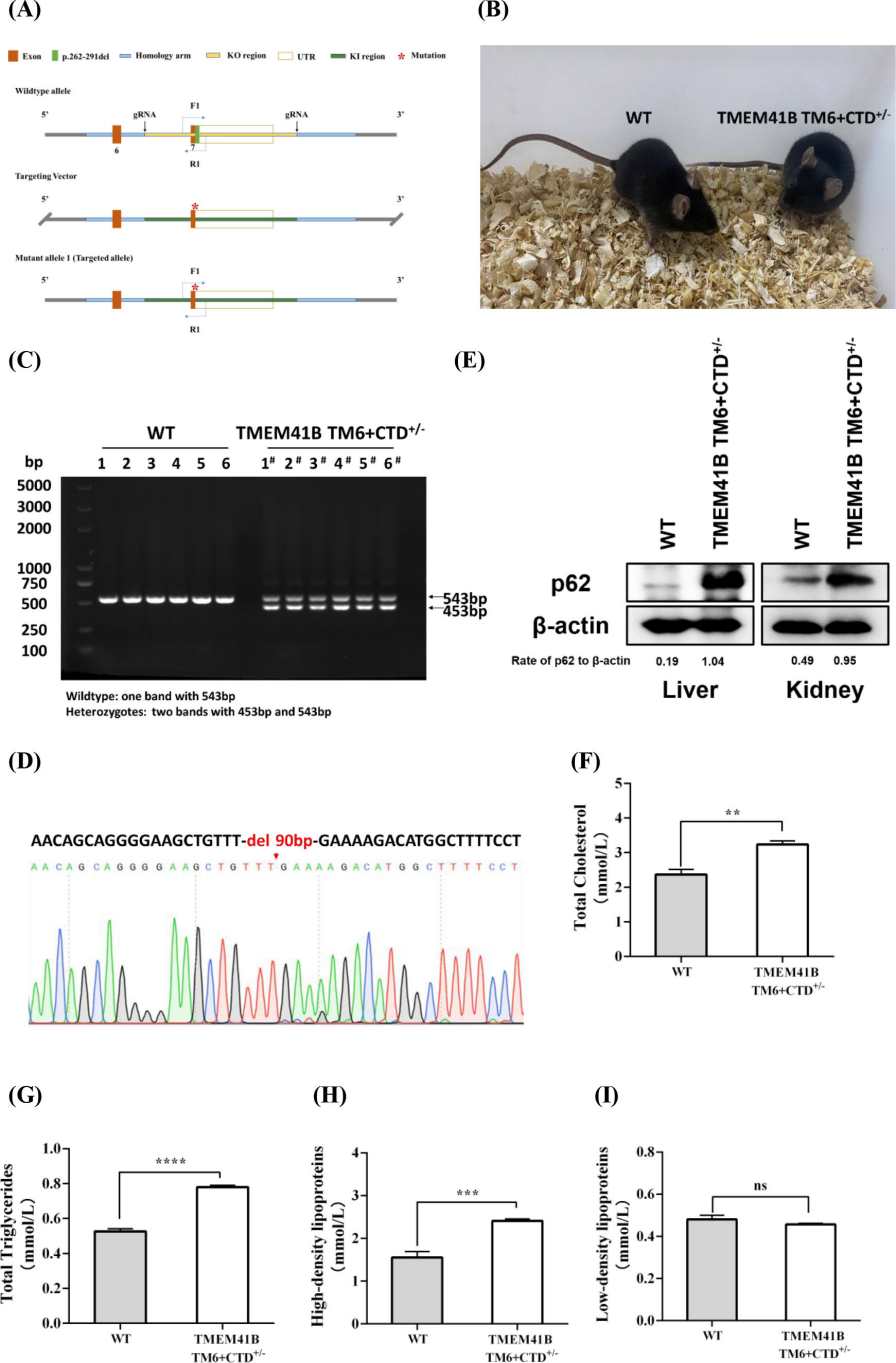
The generation and characterization of TMEM41B TM6+CTD-deficient mice. (**A**) Overview of the targeting strategy for generating TMEM41B TM6+CTD^+/−^ mice. (**B**) No obvious morphological difference between TMEM41B TM6+CTD^+/−^ and WT mice. (**C**) Identification of TMEM41B TM6+CTD^+/−^ mice via PCR amplification of genomic DNA from tail clips. WT mice were used as controls. (**D**) Sanger sequencing of PCR products from TMEM41B TM6+CTD^+/−^ mice. (**E**) Liver and kidney tissues from WT and TMEM41B TM6+CTD^+/−^ mice were subjected to Western blot assay for detecting the autophagy marker p62 protein. Target protein expression was quantitatively estimated by ImageJ software and presented as the density value relative to that of β-actin. (**F–I**) Measurement of the serum lipids, including cholesterol (**F**), triglycerides (**G**), high-density lipoproteins (**H**), and low-density lipoproteins (**I**) of WT and TMEM41B TM6+CTD^+/−^ mice (before MHV infection) via the corresponding reagent kit. **P < 0.01; ***P < 0.001; ****P < 0.0001.

To further investigate whether the deletion of the TM6 domain of TMEM41B impacts coronavirus infection *in vivo*, a mouse knock-in (KI) model in C57BL/6J mice was generated using CRISPR/Cas9 technology. To ensure the comparability of results both *in vitro* and *in vivo*, the deletion of TM6+CTD in the donor is introduced into exon 7 via homology-directed repair (Fig. 7A). However, only heterozygous TMEM41B TM6+CTD^+/−^ mice were obtained (Fig. 7B through D). We detected a notable impairment of autophagic flux, characterized by p62 accumulation, in the livers and kidneys of TMEM41B TM6+CTD^+/−^ mice compared to WT mice (Fig. 7E). However, the serum triglyceride, total cholesterol, high-density lipoprotein, and low-density lipoprotein did not decline in TMEM41B TM6+CTD^+/−^ mice compared to those in WT mice (Fig. 7F through I).

**Fig 8:**
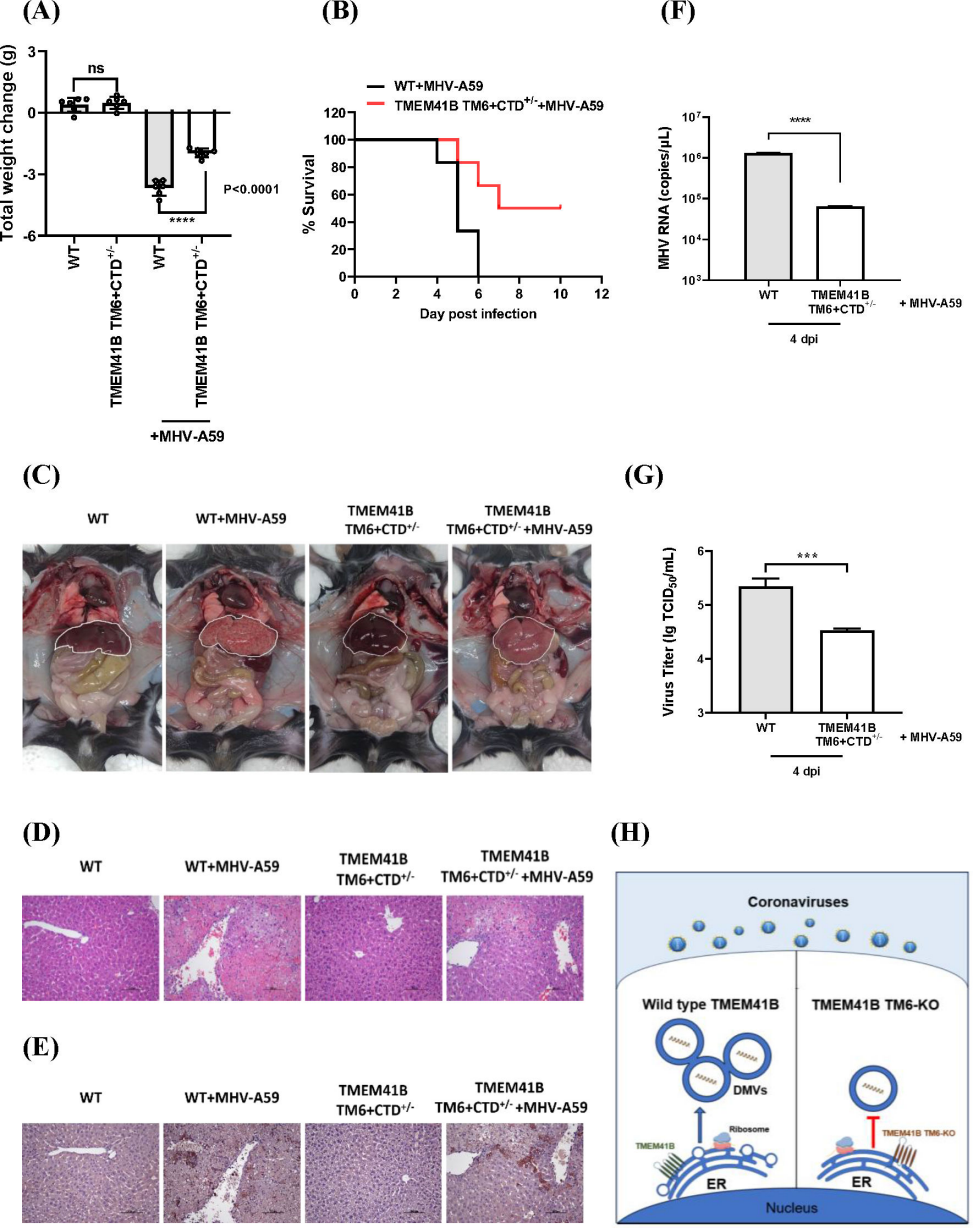
TMEM41B TM6 domain is required for coronavirus infection *in vivo*. (**A**) Total weight change of TMEM41B TM6+CTD^+/−^ and WT mice during the 4 days post-inoculation (dpi) of MHV-A59. (**B**) Survival curves for TMEM41B TM6+CTD^+/−^ and WT mice infected with MHV-A59. (**C and D**) Detection of macroscopic and microscopic lesions of the liver in TMEM41B TM6+CTD^+/−^ and WT mice at 4 dpi via necropsy examination (**C**) and histological examination (**D**) of hematoxylin and eosin staining. (**E**) Immunohistochemical analysis of the liver in TMEM41B TM6+CTD^+/−^ and WT mice for detecting MHV-A59 infection. (**F and G**) RT-qPCR (**F**) and TCID_50_ (**G**) assays for detecting viral replication in the liver of TMEM41B TM6+CTD^+/−^ and WT mice infected with MHV-A59. (**H**) A proposed working model elucidating the roles of TMEM41B TM6 domain in coronavirus replication. The deletion of TMEM41B TM6 domain reduces the formation of double-membrane vesicles (DMVs), thereby inhibiting coronavirus replication. ***P < 0.001; ****P < 0.0001.

TMEM41B TM6+CTD^+/−^ and WT mice were intraperitoneally inoculated with MHV-A59 (plaque-forming units [PFU] = 1 × 10^6^). The control groups received an equal volume of DMEM. The weight and mortality of each mouse were monitored and recorded daily. As shown in Fig. 8A, the body weights of TMEM41B TM6+CTD^+/−^ mice infected with MHV-A59 slightly declined compared to those of WT mice. Moreover, TMEM41B TM6+CTD^+/−^ mice exhibited a lower fatality rate compared to WT mice upon MHV-A59 infection (Fig. 8B). Necropsy examination showed that TMEM41B TM6+CTD^+/−^ mice exhibited an obviously alleviated liver tissue lesion caused by MHV-A59 infection (Fig. 8C). Histopathological analysis further indicated extensive necrotic areas in the livers of WT mice, while reduced necrotic foci were observed in the livers of TMEM41B TM6+CTD^+/−^ mice (Fig. 8D). Additionally, immunohistochemical staining demonstrated a reduced level of MHV N in the livers of TMEM41B TM6+CTD^+/−^ mice (Fig. 8E). Furthermore, viral RNA copies and titers were examined by RT-qPCR and TCID_50_ assays, revealing that MHV-A59 exhibited lower replication levels in the livers of TMEM41B TM6+CTD^+/−^ mice compared to those of WT mice (Fig. 8F and G). These results confirm that the TM6 domain of TMEM41B plays a critical role in the infection and pathogenicity of coronavirus *in vivo*.

## DISCUSSION

The identification of host-dependency factors not only enhances our understanding of the interactions between viruses and host cells but also guides the development of antiviral gene-edited animals. Since the advent of CRISPR/Cas9 technology, the identification and characterization of host factors associated with viral replication have been extensively reported (23–25, 29, 36). TMEM41B has garnered significant attention, and considerable progress has been made in understanding its biology in recent years (37–39). However, the underlying mechanisms and identification of key functional domains of TMEM41B that promote viral replication remain poorly understood. In this study, we confirmed that the TM6 domain of TMEM41B is required for coronavirus infection both *in vitro* and *in vivo*. Our results deepen the understanding of the structural function of TMEM41B and may guide the development of novel antiviral therapeutics.

In this study, we further determined that TMEM41B KO suppresses the replication step of PDCoV while not affecting the attachment, internalization, and release steps. This finding is in agreement with previous reports that TMEM41B functions at a stage subsequent to viral entry into cells (36, 40). However, a recent study suggested that TMEM41B is critical for the internalization and early-stage replication of TGEV (29). This implies that TMEM41B serves as a multi-functional host factor, potentially playing various roles across different viruses. Furthermore, our results demonstrated that TMEM41B KO did not obviously impact the replication of PRV, VSV-GFP, and PoRV. However, Sun et al. reported that TMEM41B KO significantly inhibited VSV replication in PK-15 cells (29). Additionally, a recent report indicated that TMEM41B knockdown significantly suppresses PRV replication (41). Another study reported that the replication of VSV and herpes simplex virus type 1 was not significantly affected by the lack of TMEM41B, which supports our findings (30). This study also indicated that TMEM41B is essential for yellow fever virus (YFV) infection in HAP1, JEG3, and A549 cells but is not required in Huh-7.5 cells (30). Moreover, we have expanded the list of coronaviruses that require TMEM41B for efficient replication to include PEDV and SADS-CoV. Collectively, these results suggest that the role of TMEM41B in viral replication is complex and varies among different viruses and cell types.

TMEM41B is localized in the ER and possesses a characteristic transmembrane domain in its central region, referred to as the VTT domain. Similar domains are found in other proteins, including VMP1, TMEM41, and yeast Tvp38 (33, 37). The VTT domain is highly conserved across species; however, its structure and function remain poorly understood. Recent studies have suggested that the VTT domain contains several transmembrane helices and two reentrant loops that face each other within the membrane, potentially playing a crucial role in lipid and solute transport (37). We predicted the structure of porcine TMEM41B-WT and its mutant TMEM41B-Δ(TM6+CTD) using trRosetta (42) and found that both TMEM41B-WT and TMEM41B-Δ(TM6+CTD) contain two reentrant loops (Fig. S1C and D). This suggests that the deletion of TMEM41B TM6+CTD may not affect the formation of reentrant loops or their potential functions. Hoffmann et al. first demonstrated that the VTT domain of TMEM41B is required to support flavivirus infection and further indicated that an SNP at amino acid position 266 influences flavivirus pathogenesis in humans (30). Our results showed that this SNP is located outside the VTT domain, specifically within the TM6 domain, suggesting that the TM6 domain may also play crucial functions. Experiments with truncated mutants provided evidence that the NTD and CTD of TMEM41B do not primarily contribute to viral infection, in agreement with a previous report (30). While TMEM41B TM6 is required for coronavirus infection, it is not essential for PRV, VSV, and PoRV, which aligns with results obtained from TMEM41B KO cells (Fig. 5). The role of TMEM41B VTT domain in the infection of other coronaviruses or viral families remains to be explored in both *in vitro* and *in vivo* studies.

In this study, we also confirmed the functional relevance of the TMEM41B TM6 domain for coronavirus infection *in vivo*. Following MHV-A59 infection, TMEM41B TM6+CTD^+/−^ mice exhibited delayed disease progression, increased survival rates, and significantly lowered viral titers compared to WT mice (Fig. 8). However, we were unable to obtain homozygous KO mice, indicating that the TMEM41B TM6 domain is essential for mouse embryonic development. Our results showed that autophagic activity is disturbed *in vivo*, unlike at the cellular level (Fig. 4 and 7E). Additionally, the serum lipid levels did not decline in TMEM41B TM6+CTD^+/−^ mice compared to those in WT mice (Fig. 7F through I). These results indicated that TMEM41B TM6+CTD^+/−^ mice may exhibit deficiencies in autophagic activity but not in lipid levels. Indeed, IFA results demonstrated that TMEM41B-Δ(TM6 +CTD) retains the ability to maintain normal LD distribution (Fig. 4B). A previous study showed that TMEM41B SNP variants I266L and I266V significantly reduced YFV infection, yet they appear to be fully functional in maintaining normal lipid distribution (30). This suggests that TMEM41B-mediated LD homeostasis may not be the principal factor contributing to efficient viral infection.

The ER-localized TMEM41B has been identified as a gene essential for early autophagosome formation and lipid mobilization (32, 43). Many positive-strand RNA viruses, including coronaviruses, extensively remodel host endomembranes to form replication organelles known as perinuclear double-membrane vesicles (DMVs). The formation of DMVs, induced by coronavirus non-structural proteins (nsp3, nsp4, and nsp6), involves various host proteins necessary for the ER-associated degradation stress response. Several studies have indicated that TMEM41B plays a crucial role in the formation of coronavirus replication organelles, thereby facilitating viral infection (24, 29, 30). For instance, Sun et al. demonstrated that TMEM41B colocalizes with coronavirus nsp3, suggesting its recruitment to participate in viral replication, potentially through interactions with nsps of coronaviruses (29). Additionally, TMEM41B has been reported to interact with non-structural protein 4A (NS4A) and NS4B of YFV and Zika virus to promote membrane curvature (30). Our results also found that PDCoV nsp4 interacts with TMEM41B (data not shown), further supporting the notion that TMEM41B’s role in DMV formation may be conserved across coronaviruses and possibly other viruses such as YFV. However, Trimarco et al. were unable to visualize any replication complexes in the absence of TMEM41B and proposed that an unknown, non-lipid mobilization role for TMEM41B prior to their formation of replication complexes may be its critical function during viral infection (36). We performed a transmission electron microscopy assay to examine the presence or absence of DMVs in PDCoV-infected TMEM41B-WT and TMEM41B (TM6+CTD)-KO LLC-PK1 cells. We observed typical DMVs (diameter ~200 nm) in TMEM41B-WT cells upon PDCoV infection. Conversely, no typical DMVs were detected in TMEM41B (TM6+CTD)-KO cells. These results indicate that the TM6 domain is required for TMEM41B to contribute to the formation of coronavirus replication organelles (data not shown). Consequently, we propose a hypothetical working model that elucidates the roles of the TMEM41B TM6 domain in coronavirus replication (Fig. 8H). We speculate that the deletion of TM6 impairs the autophagy pathway and may indirectly affect other cellular signaling pathways, leading to the disruption of DMV formation. However, this hypothesis requires further validation through extensive biological experiments. Previous studies have successfully employed CRISPR/Cas9 technology to generate pigs with a deletion of the CD163 SRCR5 domain, which conferred complete resistance to both PRRSV genotypes while preserving biological function (44, 45). Therefore, further identification and characterization of key amino acids or domains of TMEM41B that are required for viral infection but not essential for mammalian embryonic development will significantly advance the development of genetically engineered animals with broad-spectrum virus resistance.

In conclusion, our present study demonstrates that knockout of TMEM41B inhibits the replication step of PDCoV infection. By systematically dissecting the functional domains of the protein, we identified TM6 of TMEM41B as a critical domain for coronavirus infection both *in vitro* and *in vivo*. These findings enhance our understanding of the structure-function relationship of TMEM41B and provide a new perspective for the development of antiviral gene-edited animals.

## MATERIALS AND METHODS

### Cells, virus, and reagents

LLC-PK1 (porcine kidney) cells (CL-101) were obtained from the American Type Culture Collection (USA). L929 fibroblast (NCTC clone 929) cells were kindly provided by Prof. Guiqing Peng at Huazhong Agricultural University. LLC-PK1 and L929 cells were maintained in modified Eagle medium (MEM; Hyclone, USA) or Dulbecco’s modified Eagle medium (DMEM; Gibco, USA) supplemented with 10% fetal bovine serum (FBS) and 1% penicillin/streptomycin solution (Hyclone). All cells were grown at 37°C with 5% CO_2_.

PDCoV strain CHN-HN-2014 (GenBank accession number KT336560), TGEV strain WH1 (GenBank accession number HQ462571), SADS-CoV strain CHN-GD-2017 (GenBank accession number MH539766), and PEDV strain AJ1102 (GenBank accession number JX188454.1) were isolated from neonatal piglets with acute diarrhea (46). MHV A59 strain (GenBank accession number MF618253.1) is a kind gift from Prof. Guiqing Peng at Huazhong Agricultural University. PRV strain Ea, PoRV strain RHeN2 (GenBank accession numbers PV026137.1–PV026147.1), and VSV-GFP were described previously (47, 48). PDCoV, PEDV, SADS-CoV, and PoRV were propagated in LLC-PK1 cells in the presence of 7.5 μg/mL trypsin, while TGEV, PRV, and VSV-GFP were propagated in LLC-PK1 cells in MEM containing 2% FBS. MHV was propagated in L929 cells in DMEM with 2% FBS.

Mouse monoclonal antibodies (mAbs) against PDCoV S protein, PDCoV N protein, SADS-CoV S protein, PEDV N protein, TGEV N protein, PRV gB protein, and PoRV VP6 protein were prepared and described previously (28). Mouse anti-MHV N antibody was a kind gift from Prof. Zhaohui Qian at Peking Union Medical College. Anti-Flag mouse mAb and anti-p62 rabbit polyclonal antibody (pAb) were purchased from Medical and Biological Laboratories (Nagoya, Japan). Anti-β-actin rabbit pAb was purchased from ABclonal (Wuhan, China). Horseradish peroxidase (HRP)-conjugated secondary antibodies were purchased from Beyotime (Shanghai, China). Alexa Fluor 488/594 goat antimouse IgG antibodies were purchased from Abbkine (Wuhan, China). HCS LipidTOX Deep Red neutral lipid stain (1:1,000; Invitrogen, USA) was used for lipid staining.

### Western blot analysis

Cells were transfected or infected with viruses for the indicated time and then were lysed with RIPA lysis buffer (Beyotime), followed by centrifugation at 12,000 rpm at 4°C for 10 min. The clarified supernatants were collected, mixed with 5× SDS loading buffer, and then boiled for 10 min. Cellular protein samples were separated by 12.5% SDS-PAGE (Yazyme, China) and transferred onto polyvinylidene fluoride membranes (Millipore, USA). Residual binding sites were blocked with 10% non-fat milk in TBST (10 mM Tris, 100 mM NaCl, and 0.1% Tween 20) for 2 h at room temperature. Then, the membranes were probed with specific primary antibodies for 4 h at room temperature or overnight at 4°C, followed by HRP-conjugated secondary antibodies (1:5,000 dilution) for 1 h. After three washes, the membranes were visualized using enhanced chemiluminescence reagents (Vazyme, China) via ChemiDoc Touch Imaging System (Bio-Rad, Hercules, CA, USA).

### Indirect IFA

Cells grown in 24-well plates were infected with different viruses for the indicated time point and then washed twice with PBS and fixed with 4% formaldehyde for 15 min at room temperature. Treated cells were permeabilized with methanol for 10 min and blocked with 5% (wt/vol) bovine serum albumin in PBS for 1 h. The cells were incubated with primary antibodies for 1 h at 37℃ or overnight at 4℃. After three washes with PBS, cells were incubated with Alexa Fluor 488/594-conjugated goat antimouse IgG antibodies (1:2,000 dilution) at 37°C for 1 h. Cell nuclei were stained with 4ʹ,6-diamidino-2-phenylindole. The images were visualized and captured with a fluorescence microscope (Olympus, Tokyo, Japan).

### RNA extraction and RT-qPCR

Total RNAs were extracted from virus-infected cells or tissues using TRIzol reagent (Invitrogen). For cDNA synthesis, total RNA (1 µg) from each sample was reverse transcribed into cDNA using the reverse transcriptase (Vazyme). The quantitative PCR reaction was performed following the Minimum Information for Publication of Quantitative Real-Time PCR Experiments guidelines (49). A total of 10 μL reaction volume (including 1 μL of the above cDNA, 0.2 μL of specific forward/reverse primers, 5 μL of 2× Taq Pro Universal SYBR qPCR Master Mix, and 3.6 μL of RNase-free water) was subjected to RT-qPCR in an ABI 7500 real-time PCR system (Applied Biosystems, USA). For an absolute qPCR, gene mRNA levels were calculated using standard curves. Analysis of the results was done using QuantStudio Real-Time PCR software (Applied Biosystems). The negative- and positive-strand genome RNA of PDCoV was quantified at the 5′ untranslated region and N gene using the primers (q5′UTR-F/R, qPDCoV-N-F/R) reported previously (50). The qPCR primers were synthesized by Sangon Biotech Corporation (Wuhan, China) and listed in Table S1.

### Plasmid construction and generation of cell lines stably expressing foreign proteins

The full-length cDNA of porcine TMEM41B was amplified from LLC-PK1 cells using specific primer TMEM41B-F/R and then cloned into pCAGGS-Flag-N with an N-terminal Flag tag to generate pCAGGS-Flag-TMEM41B. We constructed a series of truncated mutant expression plasmids with the deletion of individual NTD, CTD, VTT, TM6, and TM6+CTD, named pCAGGS-Flag-TMEM41B-ΔNTD, -ΔCTD, -ΔVTT, -ΔTM6, or -Δ(TM6+CTD), by cloning them into pCAGGS-Flag-N. To construct cell lines stably expressing eGFP, TMEM41B-WT, and TMEM41B-Δ(TM6+CTD), we cloned the gene of TMEM41B-WT and TMEM41B-Δ(TM6+CTD) into the pEGFP-C1 vector. Resulting expression plasmids, including pEGFP-TMEM41B and pEGFP-TMEM41B-Δ(TM6+CTD), and vector control pEGFP-C1, were transfected into TMEM41B-KO LLC-PK1 cells. Subsequently, positive transfected cells were selected with 500 µg/mL geneticin (G418; Solarbio, China) and were grown in MEM supplemented with 10% FBS and 200 μg/mL G418. All primers used in the experimental study are listed in Table S1. Expression constructs were validated by Sanger sequencing.

### Generation of TMEM41B KO cell lines

sgRNAs against exon 7 of the TMEM41B gene were designed with Guide Design Web (https://zlab.bio/guide-design-resources). sgRNA #1–3 were against the porcine TMEM41B gene, while sgRNA #4–6 were against the mouse TMEM41B gene (Table S2). Synthesized sgRNAs were annealed and cloned into the PX459 vector by a restriction endonuclease BbsI according to a standard protocol, resulting in recombinant plasmids PX459-sgRNAs. To construct knockout cell lines, LLC-PK1/L929 cells in 24-well plates were transfected with 0.8 μg of PX459-sgRNA (sgRNA #1–3 for LLC-PK1 cells and sgRNA #4–6 for L929 cells) for 36 h and then selected with puromycin (2 μg/mL) for 48 h. Surviving cells were cultured and then subcloned by performing finite continuous dilution in 96-well plates for clonal expansion, followed by genomic PCR amplification and Sanger sequencing. To generate LLC-PK1 cell lines with precise deletion of the TM6+CTD region of TMEM41B (TMEM41B TM6+CTD KO), two specific sgRNAs (pTMEM41B-sgRNA-up and pTMEM41B-sgRNA-down) targeting both the upstream and downstream sequences approximately 100 bp in position of the TM6+CTD region were designed and synthesized (Table S2). According to the above description, generated PX459-sgRNAs were transfected into LLC-PK1 in the presence of a homologous DNA template, followed by selection via puromycin and monoclonal screening. A similar method was used to generate TMEM41B TM6+CTD KO L929 cell lines. All sgRNA sequences used for the generation of knockout cell lines are listed in Table S2.

### Generation of TMEM41B TM6+CTD KO mice and MHV challenge

TMEM41B TM6+CTD^+/−^ mice in the C57BL/6J genetic background were generated by Cyagen (Suzhou, China). The gRNA against the mouse TMEM41B gene, the donor vector containing the “KI region-p.262-291delSWSSVFILMVLALLSILPAIFQKQLKQKFE” cassette, and Cas9 mRNA were coinjected into fertilized mouse eggs to generate targeted knock-in offspring. The offspring genotype was identified by PCR amplification of tail genomic gDNA with primers KO-F/R (Table S2), followed by Sanger sequencing. WT and TMEM41B TM6+CTD^+/−^ mice do not exhibit any baseline developmental abnormalities, weight differences, or discernible health phenotypes, and they were subjected to retro-orbital blood collection before MHV infection and not fasted prior to blood collection. The serum triglyceride, total cholesterol, high-density lipoprotein, and low-density lipoprotein were measured by Huasin Science. WT and TMEM41B TM6+CTD^+/−^ mice (6 weeks old, *n* = 6) were challenged by intraperitoneal injection of MHV-A59 (PFU = 1 × 10^6^) diluted in DMEM. The control groups were treated with an equal volume of DMEM. The weight of each mouse was monitored daily, and the lethality was recorded. Four days after intraperitoneal infection, additional mice in the parallel experiments were euthanized to observe liver pathology.

Liver tissues were fixed in 4% formaldehyde and embedded in paraffin. The fixed liver tissues were sliced into 4 µm sections by microtome, followed by hematoxylin and eosin staining and immunohistochemistry assay for detecting the liver tissue lesions and MHV infection. To evaluate viral replication in mice livers, the tissues (0.5 g) were homogenized in 1 mL of DMEM and centrifuged at 12,000 rpm for 20 min at 4°C. Supernatants were harvested and subjected to RT-qPCR and TCID_50_ assays. To detect p62, 0.1 g liver and kidney tissues of WT and TMEM41B TM6+CTD^+/−^ mice were homogenized in 1 mL RIPA lysis buffer (Beyotime) and then sonicated. The homogenates were then centrifuged at 12,000 rpm for 20 min at 4°C, and the supernatants were collected for Western blot assay.

### Statistical analysis

Statistical analyses were performed by Student’s *t*-test or one-way ANOVA using GraphPad Prism software version 8. Differences were considered to be statistically significant when the corresponding *P* values were <0.05.

## Data Availability

The data underlying this study are available in the article and its supplemental material.

## SUPPLEMENTAL MATERIAL

Supplemental material jvi.00493-26-s0001.docx Fig. S1 to S3; Tables S1 and S2.

ASM does not own the copyrights to Supplemental Material that may be linked to, or accessed through, an article. The authors have granted ASM a non-exclusive, world-wide license to publish the Supplemental Material files. Please contact the corresponding author directly for reuse.
