# Supplementary material for: Identification of TMEM41B TM6 as a critical domain for coronavirus replication in vitro and in vivo
Source: J Virol. 2026 Jul 24;100(8):e00493-26. doi: 10.1128/jvi.00493-26 (PMC13483471; doi:10.1128/jvi.00493-26)
Supplement: Supplementary file 1 [file jvi.00493-26-s0001.docx]

**Supplementary materials**

**Figure S1.** Construction of LLC-PK1 cells stably expressing TMEM41B-Δ(TM6+CTD) and its structure analysis.

**Figure S2.** Construction of LLC-PK1 or L929 cell line with precise deletion of TMEM41B TM6+CTD.

**Figure S3.** The effect of gene knockout on baseline cell proliferation.

**Table S1.** Primers used for plasmid constructions and RT-qPCR.

**Table S2.** The sgRNA sequences used for generation of knockout cell lines.

**(A)	(B)**

**(C)	(D)**

**Figure S1. Construction of LLC-PK1 cells stably expressing TMEM41B-Δ(TM6+CTD) and its structure analysis. (A, B)** Identification of three LLC-PK1 cells individually expressing eGFP, TMEM41B-WT and TMEM41B-Δ(TM6+CTD) via observation of green fluorescence and western blot assay. (**C, D**) Structural prediction of porcine TMEM41B-WT (C) and TMEM41B-Δ(TM6+CTD) (D) by trRosetta.

**(A)**

**(B)**

**(C)**

**Figure S2. Construction of LLC-PK1 or L929 cell line with precise deletion of TMEM41B TM6+CTD. (A, B)** Multiple amino acid sequences alignment of different porcine (A) and mouse (B) TMEM41B variants. (**C**) Schematic diagram of construction of LLC-PK1 or L929 cell line with precise deletion of TMEM41B TM6+CTD.

**(A)	(B)**

**Figure S3. The effect of gene knockout on baseline cell proliferation.** (**A, B**) WT，TMEM41B-KO and TMEM41B-Δ(TM6+CTD) LLC-PK1 (A) or L929 (B) cells were grown in 96-well plates to reach 80% confluence. Cell proliferations were assessed using Cell Counting Kit-8 (CCK8) assay conducted in accordance with the manufacturer’s protocol.

**Table S1.** **Primers used for plasmid constructions and RT-qPCR.**

| Primer | Nucleotide sequence (5′-3′) |
| --- | --- |
| TMEM41B-F | GAG GAATTCATGACGAAAGGCGGAG |
| TMEM41B-R | GAG CTCGAG TCACTCAAATTTCTGC |
| TMEM41B-ΔVTT-F | ATAGAATTCATGACGAAAGGCGGAG |
| TMEM41B-ΔVTT-R | CGCCTCGAGTCACTCAAATTTCTGC |
| TMEM41B-ΔTM6-R | CGCCTCGAGTCACTCAAATTTCTGCTTCAGCTTTTTTTGAACCGCCTCCC |
| TMEM41B-Δ(TM6+CTD)-R | ATACTCGAGTCAAACCGCCTCCCCCGCAG |
| TMEM41B-ΔCTD-R | ATACTCGAGTCAGAAGATGGCTGGCAG |
| TMEM41B-ΔNTD-F | CACGAATTCGGGTCAGCAAGAATGTC |
| TMEM41B-eGFP-F | ATACTCGAGCTATGACGAAAGGCGGAG |
| TMEM41B-eGFP-R | GCGGAATTCTCACTCAAATTTCTGCTTC |
| TMEM41B-Δ(TM6+CTD)-eGFP-R | ATAGAATTCTCAAACCGCCTCCCCCG |
| qPDCoV-N-F | AGCTGCTACCTCTCCGATTC |
| qPDCoV-R | ACATTGGCACCAGTACGAGA |
| q5′UTR-F | ACAGCACTGACTACCGTAGGGTTC |
| q5′UTR-R | ATTCCACGCTCCTATGGGTATCAC |
| qMHV-N-F | ATCCCCGCTTCAGAGCAAAA |
| qMHV-N-R | GGCTGTTTGCAACCCAGAAG |

**Table S2.** **The sgRNA sequences used for generation of knockout cell lines.**

| Name | Nucleotide sequence (5′-3′) |
| --- | --- |
| sgRNA#1 | GACAAGGCGGCAGGGACGCG |
| sgRNA#2 | TATTCCTCAGCATACTCTCA |
| sgRNA#3 | GGGCGGCGGAGACCGCCTAA |
| sgRNA#4 | CACCGAAGAACATCACTCCTTATAC |
| sgRNA#5 | CACCGAAAAAATGGACACCAGTATA |
| sgRNA#6 | CACCGATCTTTGCTGCCAGGCGCGG |
| pTMEM41B-sgRNA-up | CTCATTCGTGGCAATCAAAG |
| pTMEM41B-sgRNA-down | GTCCTCATCTCAGAATTGGC |
| mTMEM41B-sgRNA-up | CTGCATCAGCTTACAACAGC |
| mTMEM41B-sgRNA-down | ACTGTCATCTCAGGATTAGC |
| KO-F | CTGTCTGTAACTCTAGATCTGATGACCT |
| KO-R | TGCAGGTGACAGCACTTCTCATA |
